# Impact of Aging and Pathologies on Human Oral Mucosa: Preliminary Investigation of Biophysical Markers from Thermal and Vibrational Analyses

**DOI:** 10.3390/biom15070978

**Published:** 2025-07-08

**Authors:** Valérie Samouillan, Camille Ober, Marie-Hélène Lacoste-Ferré

**Affiliations:** 1 CIRIMAT UMR 5085, Université de Toulouse, 118 route de Narbonne, 31062 Toulouse, France; 2 Gérontopôle-CHU Toulouse, Hôpital Garonne, 224 Avenue de Casselardit, 31300 Toulouse, France

**Keywords:** biomarkers, collagen, hydration, oral mucosa, FTIR spectroscopy, thermal analysis

## Abstract

This study first examines the potential of using Fourier-transform infrared spectroscopy (FTIR) and differential scanning calorimetry (DSC) to extract molecular and organizational markers from human oral mucosa. These indicators are then examined in relation to age and pathophysiological conditions. Oral mucosa biopsies were collected from 38 patients during surgical procedures and analyzed using FTIR and DSC-validated protocols. The patients were divided into two age groups, namely 20–40 and 70–90 years. Vibrational markers of the lamina propria and epithelium, including lipid-to-protein and collagen-to-protein ratios and lipid order, were extracted from the FTIR spectra of both layers. Hydration levels and collagen thermal stability were determined from DSC thermograms of the entire biopsy. The preliminary findings of this study, which will require further validation in a larger patient cohort, indicate a significant decrease in bound water content and collagen denaturation temperature in the older population. This suggests that oral mucosa undergoes structural dehydration and collagen destabilization with age. Further comparisons within the older group revealed links between biophysical markers of the oral mucosa and chronic or local pathologies. Patients with cardiovascular diseases exhibit altered collagen organization, while patients with diabetes display differences in the lipid-to-protein ratio and the order of lipid chains in the epithelium. Gingivitis is associated with variations in the collagen-to-protein ratio, which supports the role of inflammation in extracellular matrix remodeling.

## 1. Introduction

The National Institute of Health defines a biomarker as an indicator of normal or pathological biological processes. The most well-known biomarkers are biochemical and/or molecular parameters detected in a tissue (biopsy) or a biological fluid (blood test).

In recent years, aging research has focused on biomarker signatures to understand the physiological processes that evolve with age, age-related diseases, and the aging process itself [1].

All organs can be affected by aging, particularly the integumentary system (skin, mucous membranes, and dander). Since the integument is the interface between the body and the environment, it is directly influenced by internal and external factors, such as UV rays, pollution, and shocks. The skin (or outer integument) and mucosa (inner integument) are both epithelial connective tissues.

For elderly individuals, tooth loss is commonly addressed with removable dentures that rest on the toothless ridges of the gums, specifically on the oral mucosa. This area is constantly subjected to the forces and mechanical stress of chewing [2]. Unfortunately, wearing these dentures weakens gum tissue, a fragility that intensifies with age.

The oral mucosa is composed of two primary layers, namely the epithelium and the lamina propria [3]. The epithelium overlays the lamina propria, which is anchored to the alveolar bone via the periosteum. The epithelium acts as a crucial biological barrier, protecting against chemical and microbial assaults, and shielding the lamina propria from inflammatory reactions. As a connective tissue layer, the lamina propria’s mechanical integrity is chiefly due to its main constituent, fibrillar collagens [3]. The lamina propria’s firm attachment to the underlying bone’s periosteum is crucial, as it enables dental crowns to withstand the shearing forces encountered during chewing. The lamina propria is a richly vascularized layer that supplies the overlying epithelial cells with essential nutrients and oxygen. Beyond this metabolic support, it is a dynamic tissue containing fibroblasts, blood vessels, nerves, and immune cells, allowing it to actively participate in the body’s immune defenses, especially against inflammatory reactions [4].

Histological studies of the oral mucosa have shown that epithelial thickness decreases with age. This decrease is accompanied by a reduction in keratinization, increased bacterial permeability, reduced resistance to lesions, flattening of the epithelial and basal ridges, and decreased cell density [5].

Akimoto et al. described an increase in fiber density and a change in cell adhesion in the lamina propria layer of the oral mucosa with age [6]. Additionally, disorganization of the fibrous component has been observed, resulting in increased fiber cross-linking by non-enzymatic cross-linking processes [7]. Thus, collagen becomes insoluble and highly resistant from a mechanical aspect [8].

Furthermore, in a review article, Poser et al. describe the links between the physio-pathological mechanisms of aging and oral health. They emphasize inflammatory phenomena in periodontal diseases and systemic diseases, such as diabetes and cardiovascular diseases [9].

The proposed study focuses on the identification of molecular and organizational markers of the human oral mucosa in relation to pathophysiological conditions. The literature reports the use of IR spectroscopy as a novel and non-destructive strategy for tissue studies, enabling real-time and label-free chemical analysis. This technique has been successfully employed to extract novel biomolecular markers in biological tissues [10,11,12,13,14,15,16]. In this study, we will demonstrate how the combined use of IR spectroscopy and thermal analysis, previously employed to analyze porcine gingiva [15] and dentin [16] allow for the identification of structural changes in the oral mucosa with age and under different clinical conditions characteristic of an elderly population.

## 2. Materials and Methods

### 2.1. Biopsies Collection

This transverse, pilot, monocentric study included 38 patients separated into 2 age groups, namely a “younger” group of 5 patients aged 20–40 years and an “older” group of 33 patients aged 70–90 years.

Biopsies of fresh human oral mucosa (8 mm in diameter) were collected at the University Hospital Centre of Toulouse during surgical care. Samples of human oral mucosa were limited to one biopsy per patient. They were taken from a full-thickness mucoperiosteum flap of maxillary or mandibular attached gingiva using a biopsy punch. The biopsies were rinsed with a physiological saline solution, placed on absorbent paper, and transported to the laboratory at 4 °C. The biopsies were stored at −20 °C from the day of collection until the following morning. Prior to analysis, each sample was placed in a refrigerator at 5 °C for 10 min to gently thaw.

Age, gender, location, and medical data were collected in the Case Report Form (CRF). Local data describe oral health status, taken from the OHAT (Oral Health Assessment Tool), OHI (Oral Hygiene Index) [17] and SBI (Sulcus Bleeding Index) [18].

The OHAT (Oral Health Assessment Tool) is a validated objective, hetero-assessment scale of overall oral health status [19]. The modified OHI (Oral Health Index) is validated for evaluating plaque quantity on dental surfaces in elderly subjects. The presence of plaque is a risk factor for gingivitis [17]. The modified Sulcus Bleeding Index (SBI) is a validated tool for assessing the degree of gingival inflammation (manifested by gingival bleeding). Gingivitis, which is a local inflammation of the attached gingiva, is diagnosed when the OHI and SBI are both greater than zero.

Medical data provide information on chronic pathologies and their associated treatments. These data were collected using the Charlson Comorbidity Index in the case report form (CRF).

### 2.2. Vibrational Analysis of Both Lamina Propria and Epithelium from Oral Biopsies

The vibrational analysis was carried out using Fourier-transform infrared spectroscopy in the attenuated total reflectance mode (FTIR-ATR), which is well-suited for characterizing soft biological tissues [13], dentin [16], and saliva [20]. FTIR-ATR spectra were obtained using a Nicolet 5700 (THERMO FISHER SCIENTIFIC, Waltham, MA, USA), which was equipped with an ATR accessory (Smart Orbit with a type IIA diamond crystal with a refractive index of 2.4), a KBr beam splitter, and a MCT/B detector. For attenuated total reflectance, the penetration depth of the evanescent wave into the biopsies was estimated to be between 0.4 and 3.6 µm. This enabled the successive, non-destructive study of each biopsy layer, specifically the lamina propria and the epithelium.

The biopsies were placed on the ATR device and covered with a hermetic cap containing an “O” ring to prevent the samples from dehydrating during spectral acquisition. To obtain spectra of each layer of the oral mucosa (the lamina propria and the epithelium), the biopsy was placed on the crystal successively on both sides. Measurements were recorded in the 4000–450 cm^−1^ region of with a spectral resolution of 2 cm^−1^ and 32 accumulations. A background spectrum was recorded before each experiment and subtracted from the sample spectrum. Data were collected using Omnic 8.0 (THERMO FISHER SCIENTIFIC, Waltham, MA, USA). The spectra were then baseline-corrected, smoothed using the 11-point Savitzky–Golay filter, and normalized to the amide II band present in the 1540–1550 cm^−1^ zone.

### 2.3. Thermal Analysis of the Whole Biopsies

Thermal analysis was carried out using differential scanning calorimetry (DSC), which has been validated for characterizing biological tissues [21]. DSC measurements of entire biopsy samples were performed using a DSC Pyris calorimeter (PERKIN ELMER, Waltham, MA, USA) with an empty pan serving as the reference. Defrosted biopsies (5–15 mg) were placed in hermetic aluminum pans, sealed, and subjected to a temperature program under a helium atmosphere. First, a cooling scan was performed from 20 to −100 °C at a rate of 10 °C min^−1^. Then, a heating scan was performed from −100 to 85 °C at a rate of 10 °C min^−1^. After completing the DSC measurements, the pans were reweighed to ensure that they had been correctly sealed. Then, the sample pans were then pierced and dried to a constant mass at 105 °C for 14 h to determine the dry mass of the sample and, by difference, the total amount of water in the defrosted samples.

### 2.4. Statistical Analysis

A statistical analysis was performed using OriginPro 2023 (v. 10.0.0.154, OriginLab Corporation, Northampton, MA, USA). Quantitative values are summarized in box plots showing the median and the interquartile range. We studied the link between categorical variables, including age group (20–40 or 70–90 years) and clinical indices (local and chronic pathologies), and biophysical markers of the oral mucosa using the Student’s *t*-test, or the Mann–Whitney non-parametric test when the conditions for the Student’s t-test (normality and variance homogeneity) were not met.

## 3. Results and Discussion

### 3.1. Description of the Population

The population of 38 patients is described in Table 1.

Figure S1 shows a significant increase in the OHAT score among older patients, with a median value of 7, compared to younger patients, with a median value of 1. This suggests that oral health deteriorates with age.

### 3.2. Extraction of Biophysical Markers from FTIR Spectra and DSC Thermograms

**Figure 1:**
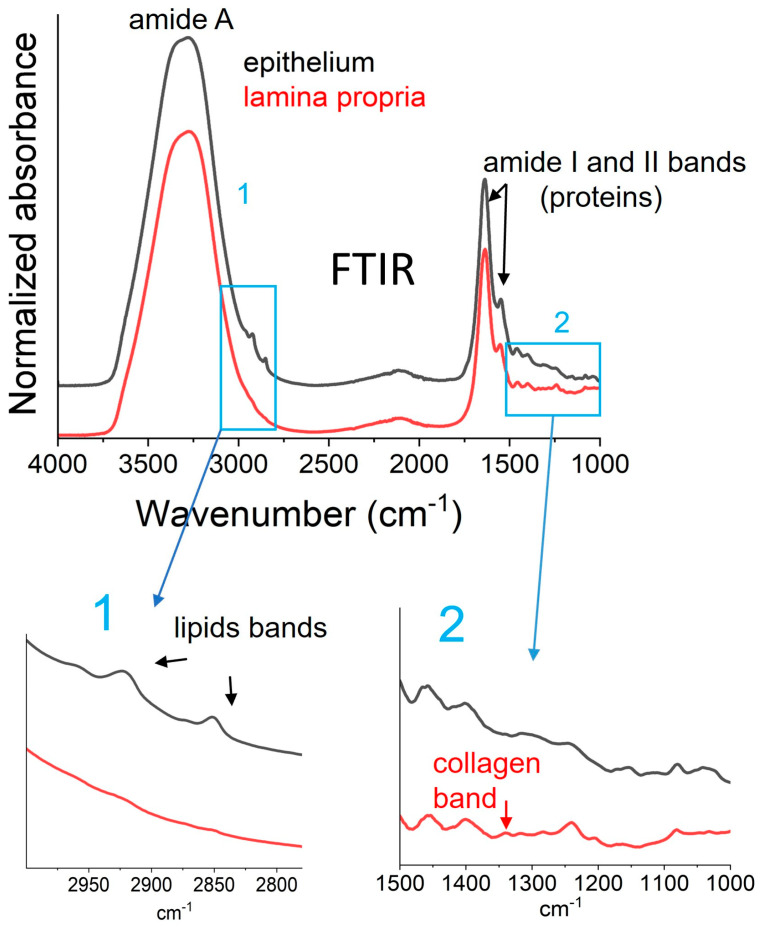
Typical FTIR/ATR spectra of lamina propria and epithelium from oral mucosa.

The typical FTIR-ATR spectra of the two constitutive layers of defrosted human oral mucosa (from a young patient) are shown in Figure 1. These spectra are similar to those of the porcine oral mucosa that we described in a previous article [15].

Notably, the intense amide A mode in the 3600–3000 cm^−1^ range is characteristic of hydrated proteins and mainly results from to the dominant absorption of free and bound water hydroxyl vibrations. In the 3000–2800 cm^−1^ region, the asymmetric and symmetric stretching modes of the (CH_2_) groups are detected at 2927 cm^−1^ and 2850 cm^−1^, respectively, in both layers. These modes are much more intense in the epithelium than in the lamina propria. These modes are primarily due to lipids and are commonly used for their quantification [22]. The distinct vibrational lipid profiles of the epithelium and lamina propria are explained by their different composition: the lamina propria is a connective tissue with low lipid content, while the epithelium is an ortho-keratinized laminated layer consisting of a stack of keratin-rich cells that accumulate lipids, such as phospholipids, ceramides, and cholesterol [15].

The amide I and II bands, which are detected in both layers within the 1800–1500 cm^−1^ spectral region, correspond to protein absorption. Unlike amide I, amide II (dominated by N-H bending coupled to C-N stretching) is not dependent on water and is often used as a normalization peak [23]. As previously observed in the FTIR spectra of human dermis [23], human periodontal tissues [14] and porcine lamina propria [15], the specific collagen absorption band at 1338 cm^−1^, which is assigned to the CH_2_ wagging of proline, is detectable only in the lamina propria. This finding is consistent with the extracellular matrix composition of the lamina propria, in which collagens account for 60% of the protein fraction.

Based on the detectable absorption bands detectable in these FTIR spectra, we identified the following vibrational indicators for this study (Table 2): the A(1338 cm^−1^)/A(Amide II) band area ratio, which is used to evaluate the collagen content in biological tissues [23], from lamina propria spectra only; and the A(2927 + 2850 cm^−1^)/A(1540 cm^−1^) band area ratio, which is used to evaluate lipids/proteins [13], from both epithelium and lamina propria spectra. Additionally, the intensity ratio I(2927 cm^−1^)/I(2850 cm^−1^), which is used as an indicator of order in acyl chains in biological tissues [12,13], was determined only from epithelium spectra. This is because the corresponding bands are low in intensity in the lamina, which could lead to an unreliable indicator.

**Figure 2:**
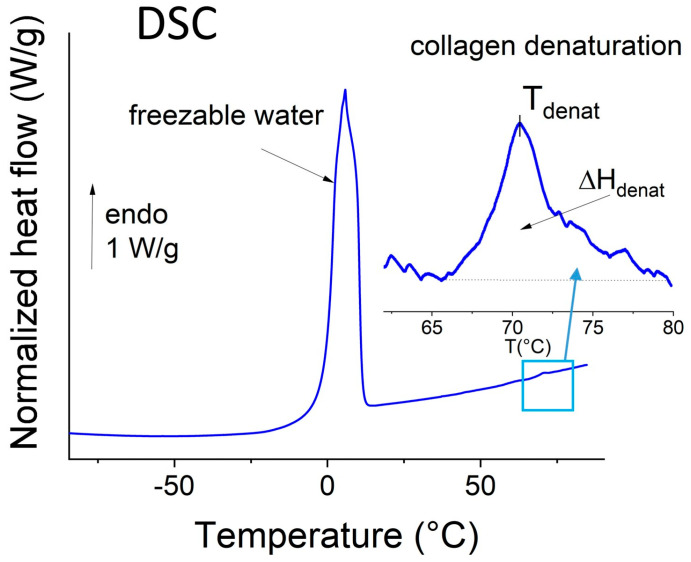
Typical DSC thermogram of human oral mucosa (whole biopsy, heating scan, 10 °C/min).

The typical DSC thermogram of defrosted human oral mucosa (from a young patient) is shown in Figure 2. This thermogram is characterized by the classical thermal events previously observed for human dermis [23], porcine oral mucosa [15], and collagen-rich tissues in general in the hydrated state [21,24].

The large endothermic peak recorded between 0 and 20 °C is attributed to the melting of frozen water. This peak is widely used to estimate the amount of free water in hydrated proteins and tissues (using the known melting enthalpy of pure ice at 0 °C). Total water content is determined by dehydration overnight at 105 °C, and the amount of bound water is calculated by difference.

Previous studies on skin [23,25], pericardium [21,26], and porcine oral mucosa [15] have shown that the endothermic peak in the 60–85 °C range corresponds to the thermal denaturation of types I and III collagen, i.e., the collapse of the triple helical structure. The temperature of the maximum is the denaturation temperature, which reflects the thermal stability of collagen [24,27]. Another thermal parameter of the denaturation is the area under the peak (ΔH_denat_), which is proportional to the net number of intra-chain hydrogen bonds in the α-helix broken during heating and is indicative of collagen structure [28]. It is normalized to the dry mass of the sample for ease of comparison. Biophysical markers related to hydration, collagen structure, and stability are also reported in Table 2.

**Figure 3:**
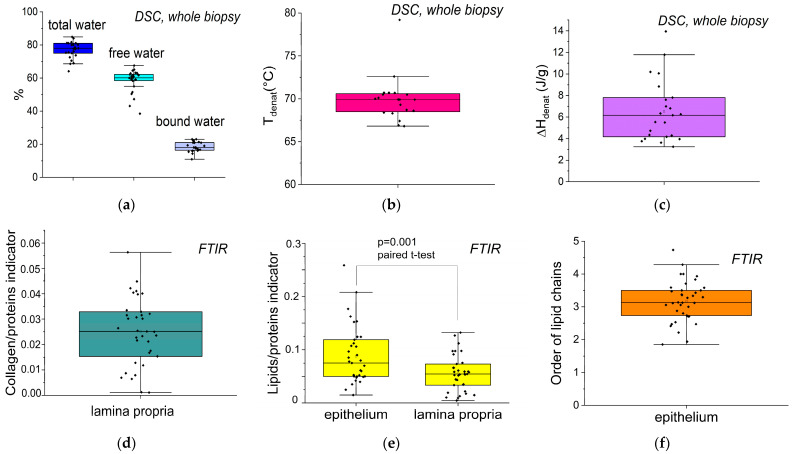
Distribution of the biophysical markers of human oral mucosa for the studied population. (**a**) Water quantification from DSC; (**b**) collagen denaturation temperature from DSC; (**c**) collagen denaturation enthalpy from DSC; (**d**) collagen/protein indicator in the lamina propria from FTIR; (**e**) lipids/protein indicator in the lamina propria and epithelium from FTIR; (**f**) order of acyl chain in epithelium from FTIR.

The distribution of these different markers was extracted for the 38 biopsies and reported in Figure 3.

The apparent dispersion observed in some indicators can be attributed to the heterogeneity of the population included in Figure 3. This figure combines data from both young and elderly patients, with or without systemic or local pathologies. Since this figure aims to provide a general overview, it only presents descriptive comparisons, without statistical testing. However, it does include a paired analysis of the lipid-to-protein ratio between the lamina and the epithelium within the same patients.

Quantification of the different types of water (Figure 3a) had not yet been reported for human oral mucosa. The total hydration level, with a median of 77%, is consistent with the value observed in porcine mucosa [15]. As in the dermis [23], this high amount of water (3.3 g/g of dry mass) is due to both structural and chemical factors. Free water (e.g., bulk water, corresponding to circulating water in the liquid phase, such as blood lymph, and water in mesopores) constitutes 3/4 of the total water, with a median value of 60%. The remaining quarter corresponds to bound water, which fills the first hydration layer of proteins [29] and other hydrophilic components of the lamina propria, such as glycosaminoglycans [30]. This water can be considered functional. No significant link was found between xerostomia and functional hydration. Xerostomia is a common side effect of medications often prescribed to geriatric patients (particularly diuretics, psychotropic, and morphinic drugs), but the prevalence of this side effect varies. For this study, we considered patients receiving treatment for chronic conditions involving diabetes and cardiovascular diseases for over two years. Some of these patients (n = 21) were also being treated for anxiety disorders and/or neuropathic pain. It could not be determined whether these anxiety disorders were transient or long-standing, which made it difficult to assess their impact on salivation.

The collagen denaturation temperature, T_denat_ (Figure 3b), with a median value of 70 °C, is similar to that reported for porcine oral mucosa [15] and hydrated collagen-rich tissues, such as pericardium and skin [21,23,24,31]. The denaturation enthalpy, ΔH_denat_ (Figure 3c), with a median of 6.2 J/g, is also similar in magnitude to that previously reported in porcine mucosa. It must be specified that the collagen/protein indicator (Figure 3d) is proportional to the collagen/protein ratio, not the ratio itself. Consistently, the indicator proportional to the lipids/protein ratio (Figure 3e) is significantly higher for the epithelium than for the lamina propria. With a median of 3.1, the lipid chain order indicator (Figure 3f) is larger than that of the brain/liver (2.4), heart (2.0), and intestine (1.77), due to the special organization of epithelial lipids.

### 3.3. Links Between Biophysical Markers and Physio-Pathological Factors

#### 3.3.1. Impact of Age

**Figure 4:**
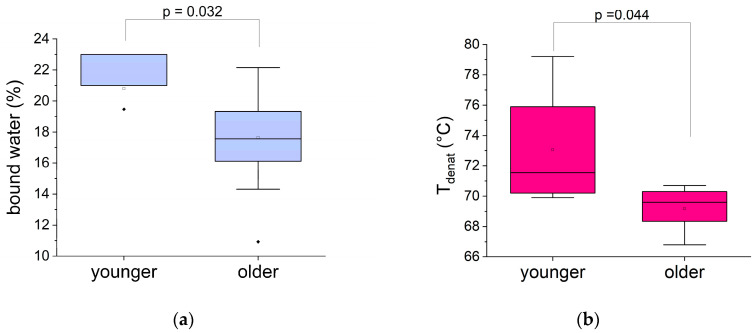
Comparison of two biophysical markers of the human oral mucosa with age: (**a**) bound water quantification; (**b**) collagen denaturation temperature.

First, we investigated the impact of age on biophysical markers. As shown in Figure 4, the amount of bound water and the collagen denaturation temperature decreased significantly with age. In contrast, there was no significant difference between the two age groups in terms of the amount of free water, the denaturation enthalpy, or the collagen/protein indicator (Figure S2).

Bound water plays a crucial role in the dynamics and, therefore, the functionality of biopolymers in biological tissues. A decrease in this marker may indicate functional dehydration of the oral mucosa in elderly patients. This is consistent with a decrease in proteoglycan monomers per hyaluronic acid molecule with age. This reduction in the overall negative charge of glycosaminoglycan chains leads to decreased water retention in the extracellular matrix [32,33]. Regarding collagen organization, its quantity remains unaffected, but its denaturation temperature decreases with age, indicating fragmentation and/or destabilization of collagen [23]. In gingival tissue, this could be associated with the inflammaging component of aging. Indeed, this population is polypathological and geriatric, often taking multiple medications, with chronic local and general inflammatory diseases. Collagen fragmentation can lead to a reduction in water bridges, which can also account for the decrease in bound water in these patients. A reduction in electrostatic bonds between type I collagen and proteoglycans has also been observed, which disrupts the integrity of the extracellular matrix [34]. These modifications corroborate the disruption of the functionality of the various extracellular matrix constituents with aging under pathophysiological conditions.

Dehydration and loss of skin softness are frequent clinical manifestations in geriatrics [35,36,37], which is consistent with these results.

#### 3.3.2. Links Between Biophysical Markers and Pathological Factors in the Older Group

For the rest of the study, we focused on investigating the links between biophysical markers and general pathologies/local clinical signs in the older population.

• Chronic pathologies

**Figure 5:**
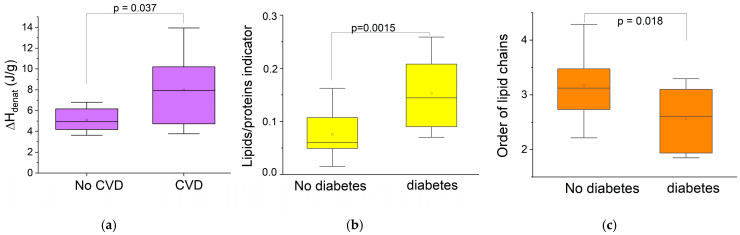
Comparison of biophysical markers of the human oral mucosa within the older group only: (**a**) collagen denaturation enthalpy for patients with and without cardiovascular diseases; (**b**) lipid/protein indicator for patients with and without diabetes; (**c**) order of acyl chains for patients with and without treated or not for diabetes.

Cardiovascular diseases and type 2 diabetes are common chronic diseases in geriatrics that impact on the oral cavity, particularly on the oral mucosa [38]. Therefore, it was interesting to look for correlations between biophysical markers and these pathologies in the group of older patients, as shown in Figure 5.

Figure 5a shows that collagen denaturation enthalpy is shown as the only oral mucosa indicator significantly impacted by cardiovascular diseases in the older group. This marker indicates a densification of collagen molecules due to an increase in hydrogen bonds in patients with cardiovascular pathologies. This modification of the extracellular matrix can be associated with the profibrotic activity observed in cardiac fibrosis and cardiovascular insufficiency [39,40].

As shown in Figure 5b,c, the two biophysical markers modified in elderly diabetic patients are related to the epithelium. The lipid/protein indicator of the epithelium is significantly higher in diabetic patients (Figure 5b). Considering that elderly diabetic patients often suffer from obesity, characterized by excess fat in tissues, it seems consistent to observe such an imbalance of the lipid component in diabetic patients [41]. More surprising is that this increase in the lipid content is accompanied by a decrease in acyl chains order in diabetic patients (Figure 5c). This is corroborated by the significant shift toward higher wavenumbers of the symmetric CH_2_ stretching band at 2850 cm^–1^ (Figure S3), which is also an indicator for probing the state of order of biological membranes [12,42]. This suggests greater motional freedom in the lipid acyl chains of the epithelium in diabetic patients.

The patients in this study were treated with either metformin or insulin for type 2 diabetes. These are two drugs with distinct pharmacodynamic effects. Metformin enhances cellular insulin action and reduces hepatic glucose release. This makes metformin the first-line treatment for prediabetes and for patients who have just been diagnosed. Metformin does not stimulate insulin secretion and, therefore, does not cause hypoglycemia. Insulin is prescribed for pancreatic dysfunction and substitutes natural insulin when secretion is insufficient. It is also used when metformin alone is inadequate. Insulin acts on insulin resistance. Metformin reduces the release of pro-inflammatory senescence-associated secretory phenotype (SASP) factors, particularly in the skin. Therefore, metformin plays a protective role in the tissues. Patients treated with insulin typically have a longer history of diabetes, leading to greater tissue damage [43]. To evaluate the effects of these treatments on epithelial biomarkers, the lipid/protein indicator was found to be higher in patients treated with insulin (mean 0.19 ± 0.06) than in patients treated with metformin (mean 0.12 ± 0.04). Similarly, acyl chain order was lower in insulin-treated patients (mean 2.20 ± 0.30) compared to metformin-treated patients (mean 2.94 ± 0.27). These findings align with the observation that diabetes complications persist despite glycemic control, driven by dyslipidemia and lipid metabolism disruptions, leading to complications, such as retinopathy, neurological disorders, renal issues, and gingival issues [44].

The literature often reports a shift toward higher temperatures for the collagen denaturation temperatures of the extracellular matrix in diabetic patients, suggesting an increase in network stability induced by protein glycation [45]. A significant dehydration of the stratum corneum has also been reported in diabetic patients due to excessive water loss [46]. However, this study does not show such an increase in collagen thermal stability or mucosal hydration in diabetic patients. This is undoubtedly due to the stabilization and regulation of blood sugar levels in treated diabetic patients.

• Local inflammation (gingivitis)

Periodontal diseases are chronic inflammatory disorders that encompass destructive and non-destructive diseases of the tissues that support teeth. Gingivitis, a non-destructive form, manifests as inflammation of the marginal gingiva and bleeding due to the accumulation of bacterial plaque. In the elderly, poor oral hygiene and immunosenescence make gingivitis widespread, particularly among dependent people suffering from cognitive diseases [47].

**Figure 6:**
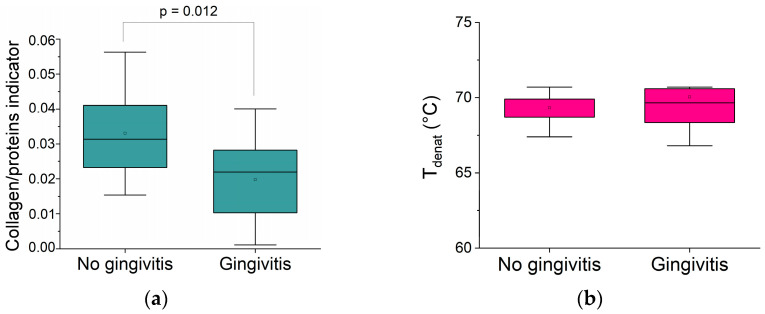
Comparison of biophysical markers of the human oral mucosa within the older group only, for patients with and without gingivitis: (**a**) collagen/protein indicator; (**b**) collagen denaturation temperature.

Figure 6a shows a significant decrease in the collagen indicator in the lamina propria of older patients with gingivitis compared to controls. In contrast, neither the collagen denaturation temperature associated with triple helix integrity (Figure 6b) nor the water organization is affected by local inflammatory signs.

The quality of the collagen phase does not appear to be altered, only the quantity. It is important to note that the biomarker modification is only observed within the inflammatory site (lamina propria) and not within the oral epithelium. A decrease in the collagen network of gingival tissue has been reported in histologic studies and has been associated with the involvement of cytotoxic cells (mainly cytotoxic T lymphocytes), which are observed during gingivitis and periodontitis [32], as well as with matrix metalloproteases [48]. A previous study also reported decreased oral mucosa stiffness of oral mucosa with gingivitis [49], which confirms the reduction in collagen and the fiber ratio in the lamina propria functionally.

## 4. Conclusions

This preliminary study shows the feasibility of using FTIR and DSC protocols to extract new biophysical markers of the hydric and molecular organization in the oral mucosa in humans. Specifically, modifications in water organization and destabilization of the collagen network have been detected with aging, though this will need to be confirmed in a larger-scale study. Additionally, thermal and vibrational markers expressed by the elderly population studied differ depending on general (e.g., cardiovascular diseases and diabetes) and local (e.g., gingivitis) physio-pathological conditions.

Future studies should focus on a larger number of healthy young and elderly individuals to understand the distribution of specific biomarkers of aging. Second, common pathologies, such as diabetes and cardiovascular diseases, should be considered in studies involving both young and elderly patients to understand their established impact on the oral mucosa. Finally, gingivitis, a common periodontal disease, should be studied in relation to age to identify specific inflammatory markers.

## Figures and Tables

**Table 1:** Description of the population including the younger and older groups.

	Younger [20–40 Years Old] n = 5	Older [70–90 Years Old] n = 33
**Sociodemographic** **characteristics**		
Age (mean ± SD)	27 ± 5	82 ± 5
Gender M/F (%)	4/1	19/14
**Oral Evaluation**		
Teeth number (mean ± SD)	30 ± 2	19 ± 8
OHAT (healthy/degraded) (%)	4/1	10/23
Gingivitis (OHI + SBI > 1) Yes/No (%)	1/4	18/15
**Chronic diseases**		
Cardiovascular pathologies Yes/No (%)	0/5	21/12
Type II diabetes yes/no (%)	0/5	6 ^1^/27

^1^ 50% of diabetes patients were treated with insulin, and 50% were treated with metformin.

**Table 2:** Biophysical markers of human oral mucosa determinable from FTIR and DSC.

	Biophysical Marker	Technique	Determination
Hydration	Free water (%)	DSC Whole biopsy	ΔH_ice melting_/334
	Total water (%)	Weighing Whole biopsy	(weight _hydrated_ − weight _dry_)/weight _hydrated_
	Bound water (%)	DSC/weighing Whole biopsy	total water(%) − free water(%)
Collagen	Thermal stability (°C)	DSC Whole biopsy	T_denat_ (from denaturation peak)
	Hydrogen bonds (J/g)	DSC Whole biopsy	ΔH_denat_ (from denaturation peak)
	Collagen/proteins	FTIR Lamina propria only	area (1338 cm^−1^)/area(1540 cm^−1^)
Lipids	Lipids/proteins	FTIR Lamina propria Epithelium	area (2927 + 2850 cm^−1^)/area(1540 cm^−1^)
	Order of lipid chains	FTIR Lamina propria only	intensity (2927 cm^−1^)/intensity (2850 cm^−1^)

## Data Availability

Data is contained within the article or supplementary material. Further inquiries can be made available on request from the corresponding authors.

## Supplementary Materials

The following supporting information can be downloaded at: https://www.mdpi.com/article/10.3390/biom15070978/s1, Figure S1: Comparison of the Oral Health Assessment Tool (OHAT) with age. Figure S2: Comparison of some biophysical markers of the human oral mucosa with age. Figure S3: Position of the CH_2_ symmetric stretching mode (from FTIR spectra) in the oral epithelium of patients with and without diabetes in the group of older patients.

## References

[1] Crimmins E., Vasunilashorn S., Kim J.K., Alley D. (2008). Biomarkers Related to Ageing in Human Populations. Adv. Clin. Chem..

[2] Chen J., Ahmad R., Li W., Swain M., Li Q. (2015). Biomechanics of oral mucosa. J. R. Soc. Interface.

[3] Waasdorp M., Krom B.P., Bikker F.J., van Zuijlen P.P.M., Niessen F.B., Gibbs S. (2021). The Bigger Picture: Why Oral Mucosa Heals Better Than Skin. Biomolecules.

[4] Cruchley A.T., Bergmeier L.A., Bergmeier L.A. (2018). Structure and Functions of the Oral Mucosa. Oral Mucosa in Health and Disease.

[5] Hamzah Z., Indartin D., Meilawaty Z. The Progressive Low Chronic Inflammation on Oral Tissue in Elderly. Proceedings of the 1st International Conference on Medicine and Health Sciences (ICMHS).

[6] Akimoto K. (2004). Observations on the Structural Changes According to Aging of Oral Mucous Membrane in the Elderly-Structure of Buccal Mucous Membrane in the Vicinity of Angulus Oris-. J. Stomatol. Soc..

[7] Quan T., Fisher G.J. (2015). Role of Age-Associated Alterations of the Dermal Extracellular Matrix Microenvironment in Human Skin Aging: A Mini-Review. Gerontology.

[8] Rajalalitha P., Vali S. (2005). Molecular Pathogenesis of Oral Submucous Fibrosis—A Collagen Metabolic Disorder. J. Oral Pathol. Med..

[9] Poser M., Sing K.E.A., Ebert T., Ziebolz D., Schmalz G. (2023). The Rosetta Stone of Successful Ageing: Does Oral Health Have a Role?. Biogerontology.

[10] Teker H.T., Ceylani T., Keskin S., Samgane G., Mansuroglu S., Baba B., Allahverdi H., Acıkgoz E., Gurbanov R. (2023). Age-Related Differences in Response to Plasma Exchange in Male Rat Liver Tissues: Insights from Histopathological and Machine-Learning Assisted Spectrochemical Analyses. Biogerontology.

[11] Rehman I., Movasaghi Z., Rehman S. (2012). Vibrational Spectroscopy for Tissue Analysis.

[12] Melin A.M., Perromat A., Deleris G. (2001). Fourier-Transform Infrared Spectroscopy: A Pharmacotoxicologic Tool for in Vivo Monitoring Radical Aggression. Can. J. Physiol. Pharmacol..

[13] Staniszewska E., Malek K., Baranska M. (2014). Rapid Approach to Analyze Biochemical Variation in Rat Organs by ATR FTIR Spectroscopy. Spectrochim. Acta A Mol. Biomol. Spectrosc..

[14] Hynes A., Scott D.A., Man A., Singer D.L., Sowa M.G., Liu K.Z. (2005). Molecular Mapping of Periodontal Tissues Using Infrared Microspectroscopy. BMC Med. Imaging.

[15] Ober C., Samouillan V., Lacoste-Ferré M.H., Dandurand J., Lacabanne C. (2020). Thermal and Vibrational Biomarkers of Porcine Oral Mucosa: Influence of Localization on Hydric Organization and Physical Structure. J. Therm. Anal. Calorim..

[16] Lauritsen A.K., Pereira J.E.M., Juranyi F., Bordallo H.N., Larsen L., Benetti A.R. (2018). Probing Water Mobility in Human Dentine with Neutron Spectroscopy. J. Dent. Res..

[17] Greene J.C., Vermillion J.R. (1964). The Simplified Oral Hygiene Index. J. Am. Dent. Assoc..

[18] Newbrun E. (1996). Indices to Measure Gingival Bleeding. J. Periodontol..

[19] Chalmers J.M., King P.L., Spencer A.J., Wright F.A.C., Carter K.D. (2005). The Oral Health Assessment Tool—Validity and Reliability. Aust. Dent. J..

[20] Simsek Ozek N., Zeller I., Renaud D.E., Gümüs P., Nizam N., Severcan F., Buduneli N., Scott D.A. (2016). Differentiation of Chronic and Aggressive Periodontitis by FTIR Spectroscopy. J. Dent. Res..

[21] Samouillan V., Delaunay F., Dandurand J., Merbahi N., Gardou J.-P., Yousfi M., Gandaglia A., Spina M., Lacabanne C. (2011). The Use of Thermal Techniques for the Characterization and Selection of Natural Biomaterials. J. Funct. Biomater..

[22] Olsztyńska-Janus S., Pietruszka A., Kiełbowicz Z., Czarnecki M.A. (2018). ATR-IR Study of Skin Components: Lipids, Proteins and Water. Part I: Temperature Effect. Spectrochim. Acta Part A Mol. Biomol. Spectrosc..

[23] Tang R., Samouillan V., Dandurand J., Lacabanne C., Lacoste-Ferre M.-H., Bogdanowicz P., Bianchi P., Villaret A., Nadal-Wollbold F. (2017). Identification of Ageing Biomarkers in Human Dermis Biopsies by Thermal Analysis (DSC) Combined with Fourier Transform Infrared Spectroscopy (FTIR/ATR). Ski. Res. Technol..

[24] Miles C.A., Avery N.C. (2011). Thermal Stabilization of Collagen in Skin and Decalcified Bone. Phys. Biol..

[25] Wiegand N., Naumov I., Nőt L.G., Vámhidy L., Lőrinczy D. (2012). Differential Scanning Calorimetric Examination of Pathologic Scar Tissues of Human Skin. J. Therm. Anal. Calorim..

[26] Samouillan V., Dandurand J., Lacabanne C., Thoma R.J., Adams A., Moore M. (2003). Comparison of Chemical Treatments on the Chain Dynamics and Thermal Stability of Bovine Pericardium Collagen. J. Biomed. Mater. Res. A.

[27] Trębacz H., Szczęsna A., Arczewska M. (2018). Thermal Stability of Collagen in Naturally Ageing and in Vitro Glycated Rabbit Tissues. J. Therm. Anal. Calorim..

[28] Giannetti G., Matsumura F., Caporaletti F., Micha D., Koenderink G.H., Ilie I.M., Bonn M., Woutersen S., Giubertoni G. (2025). Water and Collagen: A Mystery Yet to Unfold. Biomacromolecules.

[29] Kerch G., Zicans J., Merijs Meri R., Stunda-Ramava A., Jakobsons E. (2015). The Use of Thermal Analysis in Assessing the Effect of Bound Water Content and Substrate Rigidity on Prevention of Platelet Adhesion. J. Therm. Anal. Calorim..

[30] Shin J.W., Kwon S.H., Choi J.Y., Na J.I., Huh C.H., Choi H.R., Park K.C. (2019). Molecular Mechanisms of Dermal Aging and Antiaging Approaches. Int. J. Mol. Sci..

[31] Wiegand N., Vámhidy L., Lőrinczy D. (2010). Differential Scanning Calorimetric Examination of Ruptured Lower Limb Tendons in Human. J. Therm. Anal. Calorim..

[32] Radzki D., Negri A., Kusiak A., Obuchowski M. (2024). Matrix Metalloproteinases in the Periodontium-Vital in Tissue Turnover and Unfortunate in Periodontitis. Int. J. Mol. Sci..

[33] Ruiz Martínez M.A., Peralta Galisteo S., Castán H., Morales Hernández M.E. (2020). Role of proteoglycans on skin ageing: A review. Int. J. Cosmet. Sci..

[34] Reigle K.L., Di Lullo G., Turner K.R., Last J.A., Chervoneva I., Birk D.E., Funderburgh J.L., Elrod E., Germann M.W., Surber C. (2008). Non-Enzymatic Glycation of Type I Collagen Diminishes Collagen-Proteoglycan Binding and Weakens Cell Adhesion. J. Cell. Biochem..

[35] Calleja-Agius J., Muscat-Baron Y., Brincat M.P. (2007). Skin Ageing. Menopause Int..

[36] Calleja-Agius J., Brincat M., Borg M. (2013). Skin Connective Tissue and Ageing. Best Pract. Res. Clin. Obstet. Gynaecol..

[37] Camilion J.V., Khanna S., Anasseri S., Laney C., Mayrovitz H.N. (2022). Physiological, Pathological, and Circadian Factors Impacting Skin Hydration. Cureus.

[38] Patel R., Gallagher J. (2024). Healthy Ageing and Oral Health: Priority, Policy and Public Health. BDJ Open.

[39] Travers J.G., Kamal F.A., Robbins J., Yutzey K.E., Blaxall B.C. (2016). Cardiac Fibrosis: The Fibroblast Awakens. Circ. Res..

[40] Mialet-Perez J., Douin-Echinard V., Cussac D., Bril A., Parini A. (2015). Vieillissement—Une Question de Cœur?. Med. Sci..

[41] Chandrasekaran P., Weiskirchen R. (2024). The Role of Obesity in Type 2 Diabetes Mellitus—An Overview. Int. J. Mol. Sci..

[42] Klaiss-Luna M.C., Manrique-Moreno M. (2022). Infrared Spectroscopic Study of Multi-Component Lipid Systems: A Closer Approximation to Biological Membrane Fluidity. Membranes.

[43] Suda M., Paul K.H., Tripathi U., Minamino T., Tchkonia T., Kirkland J.L. (2024). Targeting Cell Senescence and Senolytics: Novel Interventions for Age-Related Endocrine Dysfunction. Endocr. Rev..

[44] Eid S., Sas K.M., Abcouwer S.F., Feldman E.L., Gardner T.W., Pennathur S., Fort P.E. (2019). New Insights into the Mechanisms of Diabetic Complications: Role of Lipids and Lipid Metabolism. Diabetologia.

[45] Melling M., Pfeiler W., Karimian-Teherani D., Schnallinger M., Sobal G., Zangerle C., Menzel E.J. (2000). Differential Scanning Calorimetry, Biochemical, and Biomechanical Analysis of Human Skin from Individuals with Diabetes Mellitus. Anat. Rec..

[46] Sakai S., Kikuchi K., Satoh J., Tagami H., Inoue S. (2005). Functional Properties of the Stratum Corneum in Patients with Diabetes Mellitus: Similarities to Senile Xerosis. Br. J. Dermatol..

[47] Lauritano D., Moreo G., Vella F.D., Stasio D.D., Carinci F., Lucchese A., Petruzzi M. (2019). Oral Health Status and Need for Oral Care in an Aging Population: A Systematic Review. Int. J. Environ. Res. Public Health.

[48] Luchian I., Goriuc A., Sandu D., Covasa M. (2022). The Role of Matrix Metalloproteinases (MMP-8, MMP-9, MMP-13) in Periodontal and Peri-Implant Pathological Processes. Int. J. Mol. Sci..

[49] Lacoste-Ferré M.H., Ober C., Samouillan V. (2023). Viscoelastic Behavior of Oral Mucosa. A Rheological Study Using Small-Amplitude Oscillatory Shear Tests. J. Mech. Behav. Biomed. Mater..

