# Supplementary material for: Impact of Aging and Pathologies on Human Oral Mucosa: Preliminary Investigation of Biophysical Markers from Thermal and Vibrational Analyses
Source: Biomolecules. 2025 Jul 8;15(7):978. doi: 10.3390/biom15070978 (PMC12292607; doi:10.3390/biom15070978)
Supplement: Supplementary file 1 [file biomolecules-15-00978-s001.zip › biomolecules-3689128-supplementary.pdf]

biomolecules

MDPI

Supplementary information

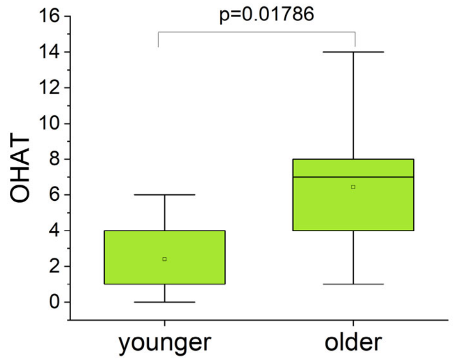

Figure S1. Comparison of the Oral Health Assessment Tool (OHAT) with age.

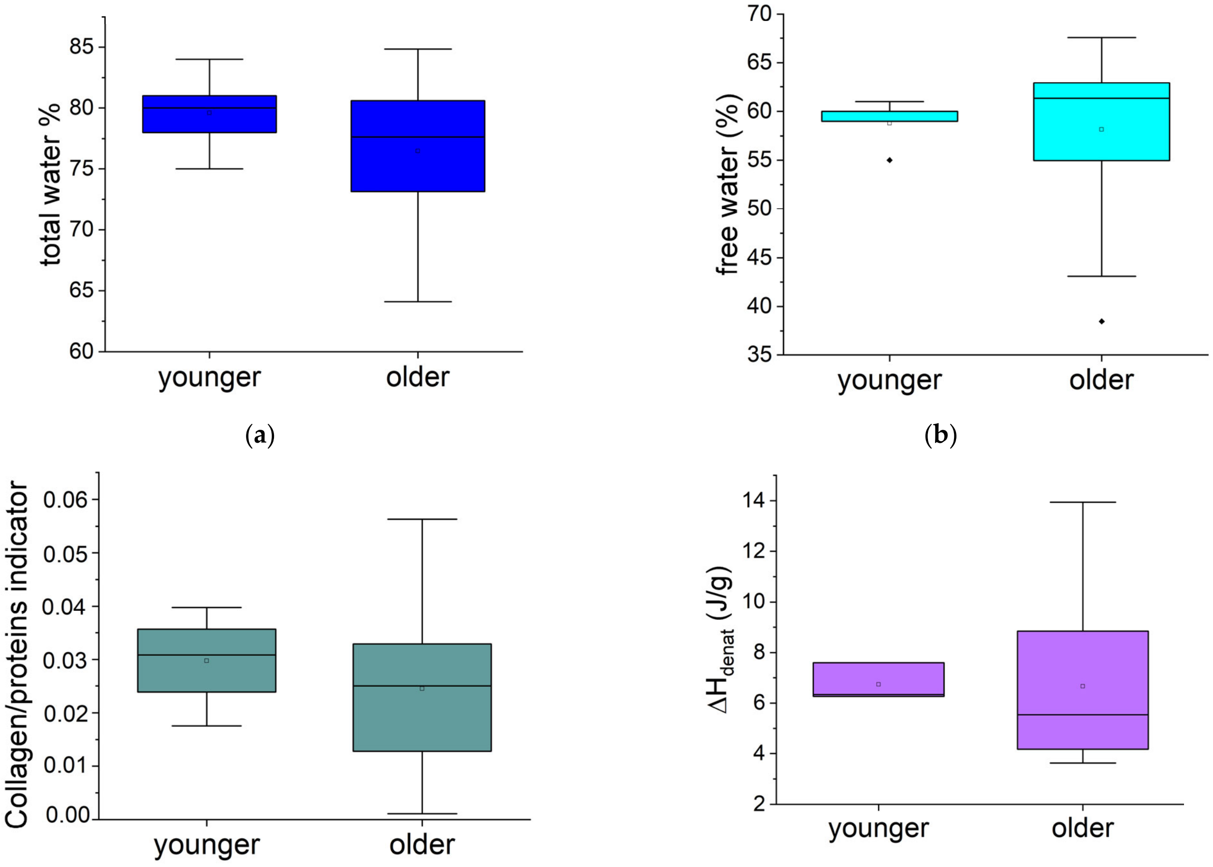

(**a**) (**b**)

(**c**) (**d**)

Figure S2. Comparison of some biophysical markers of the human oral mucosa with age: (a) Total water; (**b**) Free water; (c) Collagen/protein indicator; (d) Collagen denaturation enthalpy.

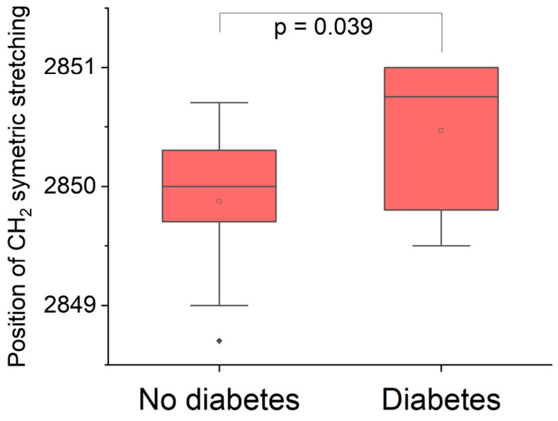

Figure S3. Position of the CH_2 symmetric stretching mode (from FTIR spectra) in the oral epithelium of patients with and without diabetes in the group of older patients.
